# NEURAP—A Dedicated Neutron-Imaging Facility for Highly Radioactive Samples

**DOI:** 10.3390/jimaging7030057

**Published:** 2021-03-16

**Authors:** Eberhard Lehmann, Knud Thomsen, Markus Strobl, Pavel Trtik, Johannes Bertsch, Yong Dai

**Affiliations:** 1 Laboratory for Neutron Scattering and Imaging, Paul Scherrer Institute, 5232 Villigen, Switzerland; knud.thomsen@psi.ch (K.T.); markus.strobl@psi.ch (M.S.); pavel.trtik@psi.ch (P.T.); 2 Laboratory for Nuclear Materials, Paul Scherrer Institute, 5232 Villigen, Switzerland; johannes.bertsch@psi.ch (J.B.); yong.dai@psi.ch (Y.D.)

**Keywords:** neutron-imaging, radiography, SINQ—Swiss spallation neutron source, NEUTRA, radioactive specimen, nuclear fuel, NEURAP, spallation target, zircaloy, lead

## Abstract

NEURAP is a dedicated set-up at the Swiss neutron spallation source (SINQ) at the Paul Scherrer Institut (PSI), optionally implemented as a special configuration of the neutron-imaging station NEUTRA. It is one of very few instrumentations available worldwide enabling neutron-imaging of highly radioactive samples to be performed routinely, with special precautions and following a specific procedure. Since the relevant objects are strong γ-sources, dedicated techniques are needed to handle the samples and to perform neutron-imaging despite the radiation background. Dysprosium (Dy)-loaded imaging plates, effectively made sensitive to neutrons only, are employed. Neutrons are captured by Dy during neutron irradiation. Then the imaging plate is erased removing gamma detections. A subsequent relatively long self-exposure by the radiation from the intrinsic neutron-activated Dy within the imaging plate yields the neutron-only radiograph that is finally read out. During more than 20 years of NEURAP operation, images have been obtained for two major applications: (a) highly radioactive SINQ target components were investigated after long-term operation life; and (b) spent fuel rods and their cladding from Swiss nuclear power plants were characterized. Quantitative analysis of the image data demonstrated the accumulation of spallation products in the lead filled “Cannelloni” Zircaloy tubes of the SINQ target and the aggregation of hydrogen at specific sites in used fuel pins of power plants and their cladding, respectively. These results continue to help understanding material degradation and optimizing the operational regimes, which might lead to extending the safe lifetimes of these components.

## 1. Introduction

SINQ is the Swiss neutron source at the Paul Scherrer Institut (PSI) for research purposes, based on spallation in a lead target that is irradiated by a 590 MeV proton beam with about 0.85 MW power from a two-stage ring-cyclotron. It is one of the large-scale facilities at PSI and provides the high-intensity neutron beams for many research instruments serving a huge user community. 

SINQ is equipped with several instrument stations for neutron-imaging with thermal (NEUTRA) [1] or cold neutrons (ICON) [2]. NEUTRA offers a special set-up dedicated to investigations of highly radioactive samples, called NEURAP.

**Figure 1:**
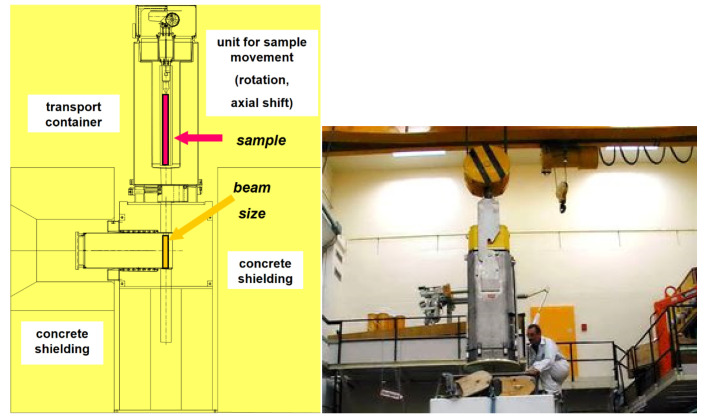
Principal set-up of NEURAP with access for the dedicated cask via the roof of the NEUTRA bunker (**left**), actual handling with the loaded transport container in PSI’s hotlab (**right**) [3], copyright permission by Elsevier, 2003.

**Figure 2:**
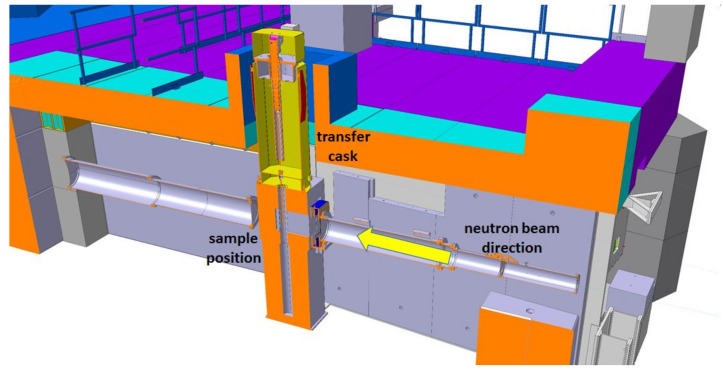
NEURAP layout inside the NEUTRA bunker. The imaging plate detector is deposed in about 20 mm from the sample; the L/D ration at this position is on the order of 350.

Inserted at the intermediate second measurement position of NEUTRA the field of view for NEURAP is approximately 6 cm wide and 20 cm high and is suitable for pin-type cylindrical rods. For highly radioactive samples, special precautions regarding shielding, encapsulation (avoidance of contamination risks) and handling equipment are required. A heavily shielded cask is used for the transport of activated samples from a hot cell. For the purpose of inserting the sample in the measurement position, the cask can be docked onto the roof of the bunker shielding of the imaging instrument NEUTRA. The highly active items to be investigated, can be coarsely pre-aligned when lowered from the cask to be well positioned with respect to the incident neutron beam. Figure 1 shows a schematic overview of the cask together with a photographic impression of the set-up and handling process. Figure 2 is an overview scheme about the real installation in 3D.

## 2. Possibilities for Investigations of Highly Radioactive Samples

The neutron-imaging instruments at PSI have been designed for flexibility with various options concerning e.g., field of view, detector, spatial and temporal resolutions. This allows for optimized working procedures with rather wide range of different specimens and for tuning the set-up to specific requirements of different applications. An important set of application cases focusses on samples with intrinsic radioactivity. Accordingly, NEURAP has been developed for the most active samples to be investigated.

### 2.1. Special Boundary Condition for the NEURAP Facility

Shielding is the most important issue to be tackled carefully when working with radioactive samples. It must be guaranteed that the dose rate limits are never exceeded during handling, transport, and observations. 

A principal challenge for neutron-imaging stems from the fact that the high level of radiation originating from the sample itself easily floods (or even damages) any detector [4], which is to acquire an image based on the incoming neutrons only. Therefore, no direct and simple exposure-mode for using neutron attenuation contrast of such sample is possible with scintillator/camera-based neutron-imaging detector technology. 

### 2.2. Principle Procedure Employing Dysprosium-Based Detection

The main problem for imaging of highly radioactive samples is to get rid of the background events caused mainly by gamma radiation from the sample itself (on the order of several 100 mSv/h in 10 cm distance) in a clever technical way. This background by far overwhelms useful counts stemming from the neutrons during the radiography exposure. 

**Figure 3:**
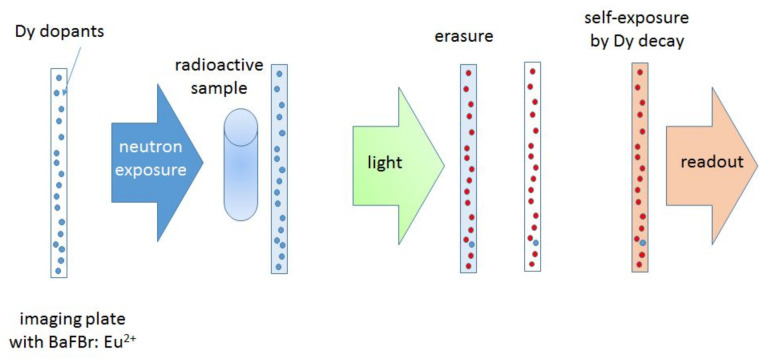
Principal steps employing activation of Dy by neutrons: the imaging plate (IP) with the Dy exposed with the radioactive sample in front (overexposure by gamma radiation allowed); all signal is removed by bright-light erasure; the decay activity from Dy-165m return the “copy” of the neutron radiography image.

The procedure used in NEURAP consists of sequential steps that lead to suppressing of the gamma background while retaining the useful neutron-imaging signal. For this, the extended temporary activation of Dysprosium by neutrons is used. The specific method [5] is schematically presented in Figure 3 and consists of the following sequential processes:

- In a first step, the sample is exposed to the neutron beam and a special imaging plate (IP) containing Dysprosium as neutron sensitive element (converter) is used for transmission imaging detection. The events generated in the IP are dominated by the gamma background of the sample. However, during an exposure of about 30 min at a flux of approximately 1E7 cm^−2^ s^−1^ neutrons are captured by Dy-164 (natural abundance 28.1%) in the IP. Dy-165 thereby becomes activated.
- After the exposure, all direct events (excitations by the gamma background) in the IP are erased by illumination with suitable bright light using a special eraser device.
- In the next step, a long self-exposure of the IP to the activity of the neutron-activated Dy-165 generates the neutron-only radiographic image.
- In the last step, finally, this indirectly neutron activation generated image is read out digitally by means of a laser IP-reader.

### 2.3. Current Methods and Possible Future Enhancements

Early neutron-imaging techniques employed X-ray films with Dy or Indium converters (after activation and transfer) or nitro-cellulose films with a B-10 converter (track-etch method).

State of the art as performed with NEURAP is using Dy-doped imaging plates employing the slow activation channel of Dy-165m (as described below). The advantage of this method lies in the high linearity of the data, enabling rather precise quantification, the direct digital output, and the option for reusing the IP after decay of the residual activation (half-life: 2.35 h; different gamma-lines contribute).

**Figure 4:**
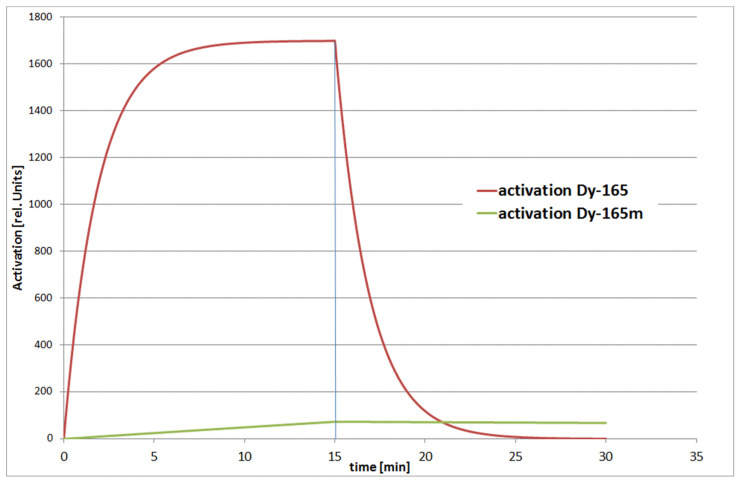
Two different channels for activation and decay of Dy-165. In the case of this IP method, only the long-living Dy-165m decay was used.

Dysprosium 165 can be activated via two different channels, i.e., Dy-165 and Dy-165m, see Figure 4. The decay times are: 1.26 min for Dy-165 (the fast channel) and 2.33 h for Dy-165m (the slow channel); see Figure 4. The associated neutron capture cross sections are 1700 barn and 1000 barn, respectively. 

With current methods and using imaging plates, only the slow channel of Dy165m is used for imaging. By the time the background produced from the gamma radiation vanishes, also the activation of the fast channel Dy165 has decayed. With this method, however, only images of static samples can be taken and obtaining a full tomography with a high number of projections is not reasonable. 

Nevertheless, attempts for tomography with a limited number of sample projections have been performed, also using the symmetry of cylindrical objects and accepting a coarse spatial resolution of sample features. 

## 3. Applications of a Facility Like NEURAP

The principal advantages of neutron-imaging fit well with the use of the method for highly radioactive samples. The usual specimens at NEURAP are made of diverse metals, which are relatively transparent for neutrons (e.g., Pb, Zr, Fe, U, …). Below, two examples of the main fields of application are introduced. These fields are:

- Studies of nuclear fuel and fuel cladding:
  ◦ Because the attenuation of U-235 differs much from U-238, the enrichment and the burn-up in fuel pins can be studied quantitatively and with high spatial resolution (pixel size 25 µm)
  ◦ Fuel claddings (mostly Zr) tend to accumulate hydrogen, which can be quantified by means of neutron-imaging; hydrogen provides a much higher neutron beam attenuation than Zr.
- Studies of SINQ spallation target components supports:
  ◦ long-term target development and safety
  ◦ quality assurance of manufactured target components
  ◦ post-irradiation investigation of critical target components and failure mode analysis
- Material tests for future potential target materials by long-term proton exposure and subsequent NEURAP investigation (e.g., STIP program)

With a power density one order of magnitude higher than in a typical nuclear reactor, SINQ is in high demand of NEURAP capabilities to understand material modifications during service life with high proton, gamma, and neutron exposure. 

All the above exemplary investigations are routinely performed at SINQ. The option of direct comparisons between fresh/un-irradiated specimen and their conditions after severe use in a highly activated state, opens unique avenues for research and development.

### 3.1. Nuclear Fuel Related Questions

**Figure 5:**
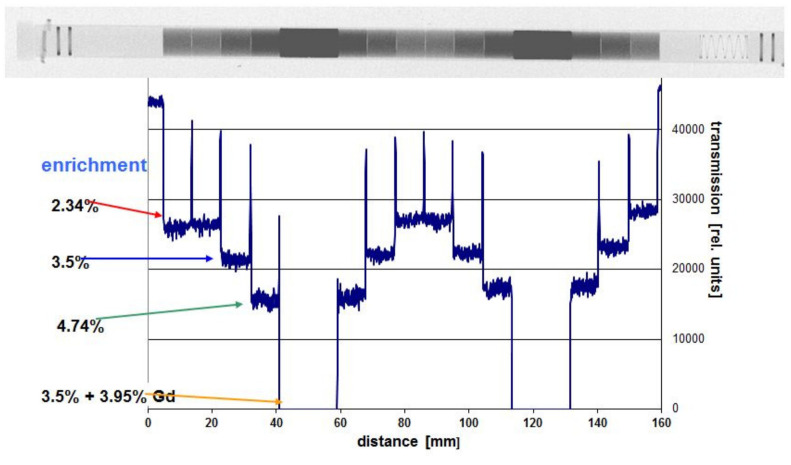
Transmission contrast and enrichment along a fuel pin also containing two sections with Gadolinium as a “burnable poison”. The outer diameter of the cylindrical samples is approximately 10 mm, copyright permission by Elsevier, 2003 [3].

**Figure 6:**
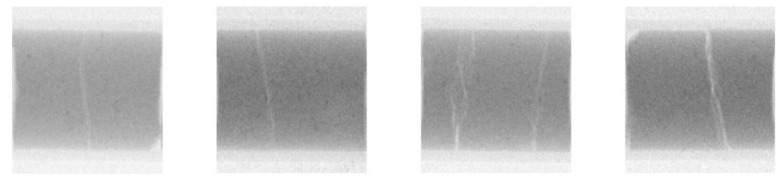
Cracks in fuel pellets. The pellets are enclosed in zirconium-based tubes of approximately 10 mm outer diameter.

**Figure 7:**
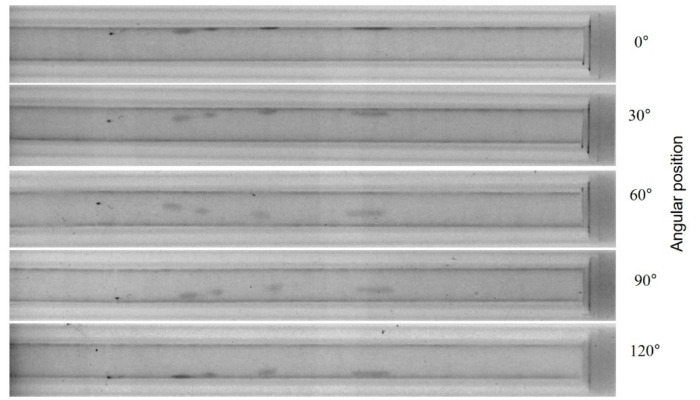
A fuel cladding tube at different rotation angles; darker areas showing the higher local hydrogen content in the cladding. The outer diameter of the cylindrical tubes is approximately 10 mm.

Figure 5, Figure 6 and Figure 7 show three different results of investigations of nuclear reactor components, where all relevant questions are readily answered by neutron-imaging, in particular, by imaging of highly activated samples after their use in nuclear reactor environments. 

The data in Figure 5 are obtained with standard imaging plates because the fuel rod is filled with un-irradiated uranium oxide pellets of different composition and enrichment of the isotope U-235. It serves demonstration purposes, however, for potential applications to irradiated fuel investigations. Some of the pellets contain also amounts of Gd, which is used as “burnable poison”. During neutron exposure in the reactor, the amount of Gd is reduced by capture and conversion into a less absorbing material. This way, the fuel criticality, which is very high at the beginning of irradiation in the reactor and decreases with the number of fissions, can be flattened for long-term operation. Thus, the performance of fuel rods in a power plant can be more evenly distributed. In the neutron images, the virgin Gd-containing pellets are completely “black”. The profile along the pellets is used to determine the enrichment completely non-invasively and with high precision, hardly possible at this level with other methods. 

Figure 6 shows some pellets of fuel elements after use in operation of a power plant. Due to the high operational temperature, temperature gradients in the reactor core, these ceramic structures are subject to stresses, can break and cracks develop. Adhering to the standard defense-in-depth strategy, all pellets are well encapsulated in the fuel rod cladding. Still, details are important to know for quality assurance purposes.

In addition, the fuel cladding undergoes material changes during reactor operation, and this can be studied by means of neutron-imaging. The images in Figure 7 are taken of an empty fuel rod, where the pellets have been removed to study the cladding behavior alone without interference from the strongly radioactive and significantly neutron attenuating nuclear material content. All darker regions contain hydrogen in varying amounts. This was also verified in destructive tests in PSI’s hotlab. By rotation of the tubes, the positions and the distributions of “hydrogen lenses” (characteristic small circular accumulations) can be observed very precisely. When the amount of hydrogen is too high, there is the risk of material embrittlement and failure of the cladding when it is mechanically loaded.

Although the investigations in Figure 5 can be performed with standard neutron-imaging methods, the images in Figure 6 and Figure 7 required the NEURAP set-up to be measured. The dose rate of the samples is approximately some 100 mSv/h in 10 cm distance. The quality of the images is comparable and sufficient for quantification, e.g., pellet imperfections and high number of cracks, or determination of the absolute amount of hydrogen and its distribution in the fuel cladding.

### 3.2. SINQ Targets

**Figure 8:**
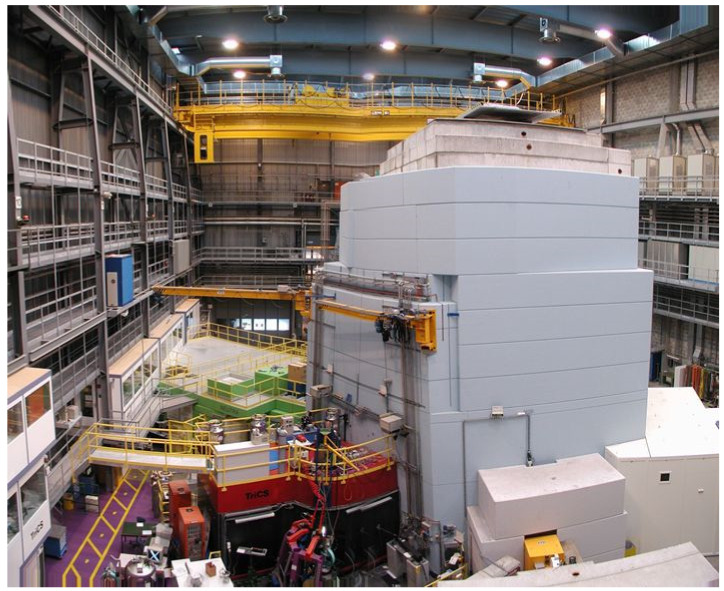
Overview of the SINQ installation; the green area on the left side is the NEUTRA imaging facility, where the NEURAP experiments have been performed, NEURAP is placed from the roof.

SINQ is a complex large-scale facility, driven by a strong proton accelerator [6]. Its dimensions are visible in Figure 8. This accelerator delivers 590 MeV protons of 1.2 mA current, corresponding to about 0.85 MW power—continuously, not in pulsed mode as other sources do. 

One of the key elements is the target in the center. It consists of a bundle of target rods (closed Zircaloy tubes with a lead filling, so-called “cannelloni”) arranged perpendicular to the proton beam direction, which is impinging vertically from below. The tube material is practically identical to the cladding of nuclear fuel rods (outer diameter 10.05 mm, wall thickness 0.606 mm), which have demonstrated their long-term stability during neutron exposure in commercial nuclear power plants.

After its operational start in 1997, the spallation target has been continually improved and tuned for highest possible neutron yield per incident proton [7]. This was supported by ongoing material qualification (e.g., with STIP probes [8]) and monitoring of the relevant material parameters. 

With the option to investigate the target rod structure before and after proton exposure (a SINQ target operates typically two years, which corresponds to an integral proton charge of 10 to 13 Ah), by means of NEURAP studies, it becomes possible to use “own” neutrons for investigations of respective components. These studies assist the safe and reliable beam time operation of the source. 

**Figure 9:**
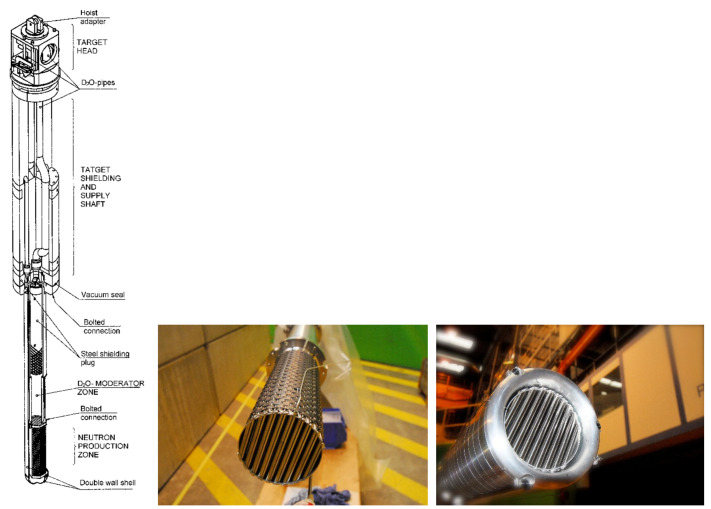
The whole SINQ target structure (**left**) and the reaction zone, a round rod bundle with Pb filled Zircaloy tubes (**middle**), surrounded by a Pb reflector blanket (**right**). The target diameter is about 20 cm.

The target rods (about 300) are arranged in a cylindrical matrix to ensure good heat removal conditions for the cooling water passing through while providing a maximum neutron output. Details are shown in Figure 9 during the assembly. 

#### 3.2.1. Quality Assurance of “Virgin” Target Rods

**Figure 10:**
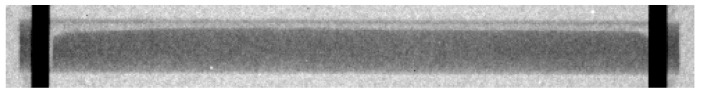
“Virgin” SINQ target rod (outer diameter approx. 10 mm) as imaged with thermal neutrons before implementation in the target structure.

A SINQ target rod consists of lead in a Zircaloy tube. Because some of the lower central rods are regularly heated by the proton beam to beyond the melting temperature of lead (327 °C) with the corresponding volume expansion, the tubes have a lead filling of only 90%. The preparation of the rods is performed by inserting solid Pb cylinders with the correct volume first, which are then subsequently heated above the melting point in an oven while keeping them in a horizontal position. This way, an empty horizontal gap is formed in the top region of each rod (see Figure 10).

Neutrons can penetrate Pb and Zr easily. Therefore, neutron-imaging is a very appropriate quality control tool for the above-mentioned procedure. For each new target, the internal Pb distribution in all 300 target rods are checked by neutron-imaging before the target assembly. If the void space above the Pb level is not equally distributed, the melting procedure of such a rod is repeated. 

The neutron-imaging investigations of these non-irradiated samples are performed using standard tools such as conventional imaging plates or camera-based systems [9]. The NEURAP system is not required here. 

#### 3.2.2. Study of the Irradiated Target Tubes

During its two-year long operation period and its high exposure to nearly one MW proton beam, the target rods are subjected to many thousands of thermal cycles because many proton beam interruptions from full power to zero power within seconds occurs during this time period. These so-called beam trips are happening typically 2–3 times per hour. In such cases, the proton beam is quickly (ms) switched off and, subsequently, ramped up again. 

Another reason for beam interruptions in SINQ is created by the synergetic use of the intense proton beam by PSI’s second target station, the ultra-cold neutron source (UCN) [10]. The proton beam is regularly (typically every 300 s) guided for a short period of time (a few seconds) towards the UCN target causing an interruption of the proton beam towards SINQ. Each of the beam interruptions causes a thermal cycle which causes an alternate melting and solidification of lead filling in some of the target rods.

##### Characterization of SINQ Targets at Different Stages of Their Lifetime

Designed for high neutron yield, a very high power density is desired. However, compared to nuclear fuel, were for which the operation of a reactor is limited by the U-235 burn-up and the accumulation of poisoning fission products, there is not such limitation in a spallation target. Nevertheless, spallation products are produced, which are mostly radioactive and, in some cases, gaseous. Prominent examples of such gases are Xe, Kr, Ar, He, and H released in certain amounts. The tight target rod (“cannelloni” tube) can withstand more than 800 bar of internal gas pressure. 

**Figure 11:**
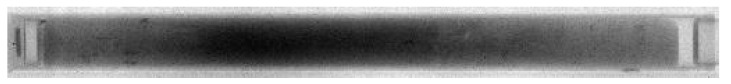
Neutron image of a target rod (outer diameter approx. 10 mm) after long-term exposure to the proton beam; the region with higher attenuation of neutrons in the center corresponds to larger amounts of spallation products with their higher absorption (view in beam direction); the dose rate in 10 cm distance and after about 1 year of decay time is still in the order of several 100s mSv/h.

Solid spallation products have often a higher neutron absorption probability and can be visualized by means of neutron-imaging studies. An example is shown in Figure 11. Wide varieties of spallation products are created in different amounts. It is impossible to attribute them directly to the proton beam profile measured of a rod, corresponding to the amount of spallation reactions. However, mercury was found to have contributed mostly to the measured contrast in the neutron image in Figure 11, and it traces the profile of the proton beam incident on the target.

**Figure 12:**
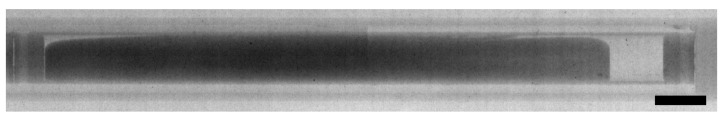
Change of the Pb distribution in SINQ target rod (outer diameter approximately 10 mm) due to the irradiation during operation oriented as inside the SINQ target; compared to the virgin status (see Figure 9) there is an accumulation in the center and ensued filling of the gap to the cladding. The scale bar corresponds to 10 mm.

Another effect during proton exposure is related to changes of the mechanical properties of the target material inside individual cannelloni tubes. For some of the central cannelloni rods with the highest heat load, the Pb filling undergoes cyclic melting-freezing transitions. It was shown by simulation experiments [11] that a redistribution of the Pb material compared to the initial filling can happen on the long-term scale even without irradiation. Inside the target, an effect comes to bear, which has been observed previously, i.e., former non-wettable materials such as stainless steel or Zircaloy are readily wetted by liquid metal under the influence of radiation. This most likely leads to a redistribution of the lead filling to the center of a target tube, leaving in the end no free “cushion”-volume for thermal expansion in radial direction in this region (see Figure 12). 

In a few cases, some target rods have consequently failed. The study of such failed components with the help of NEURAP was instrumental in investigating the effect and directing efforts to improve the target technology for the future. Avoiding these conditions altogether, in the current SINQ target a few rods in the high proton flux region have been filled by solid Zircaloy rods replacing the cannelloni rods. 

**Figure 13:**
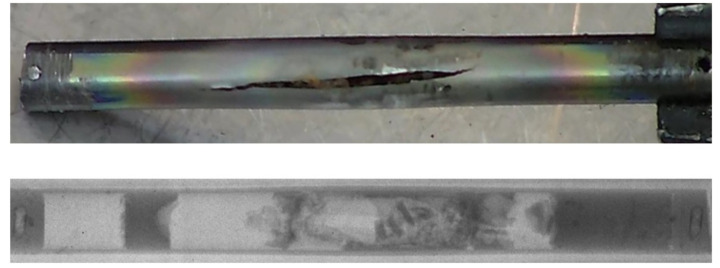
Photograph and radiography of damaged target rod (outer diameter approximately 10 mm) with remaining lead filling.

In rare cases when problems occurred with SINQ targets, NEURAP provided most valuable means for detailed investigations into failure mechanisms. Examples from the long life of SINQ are given in Figure 13.

It is important to note that in all cases where cannelloni were damaged this only effected the first barrier in the SINQ approach of defense in depth; no material was released to the outside of the save target enclosure.

### 3.3. STIP and Irradiation Programs

Spallation neutron sources are quite complex and expensive, in particular when operating close to the high power limits. Therefore, there are only a few such facilities built worldwide such as SNS, LANSCE (USA), ISIS (UK), JPARC (Japan) and CSNS (China). The source with the highest envisioned power will be ESS (Europe) in Lund (Sweden) which is presently under construction. 

**Figure 14:**
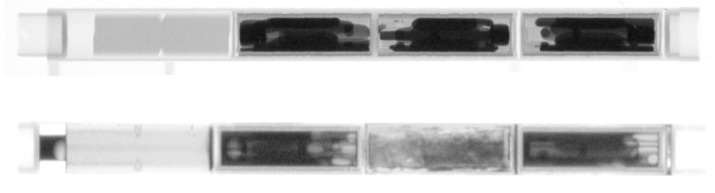
Test rod with steel encapsulating Hg samples before (**top**) and after (**bottom**) long-term exposure inside the SINQ target; the capsule in the middle has broken and Hg is leaking inside a separate enclosure, “creeping” into the corners due to the high wetting after irradiation; contact to the Zircaloy tube was successfully avoided in the other two probes.

**Figure 15:**
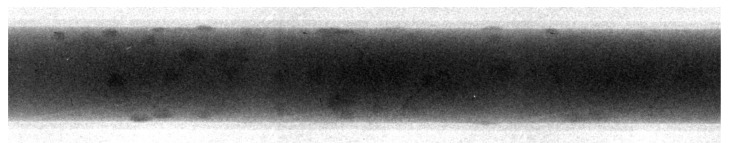
A full steel test sample with Zr cladding (diameter about 10 mm) after long-term irradiation in SINQ target 3; the black dots are interpreted as hydrogen accumulation at the outer side of steel towards the Zr cladding. Copyright by Taylor & Francis, 2001 [1].

Striving for highest power and neutron yield means also high demands on the used target materials. Although Pb is advantageously used at SINQ, mercury (Hg) (see a test sample in Figure 14) and tungsten (W) are prominent target materials in pulsed spallation neutron sources. Not only the target materials are exposed to damage during exposure, also cladding and structural materials need to be studied in detail before (and after) implementation (see, e.g., Figure 15). The understanding of materials behaviors in intensive spallation irradiation environments is essential for R&D of high power targets as mentioned above.

**Figure 16:**
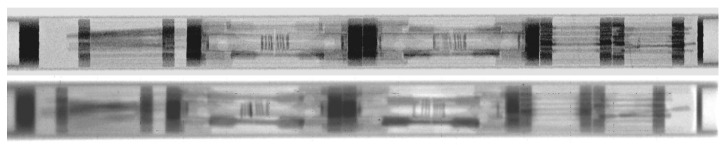
A test rod with steel specimens in encapsulated PbBi-eutectic before (**top**) and after (**bottom**) exposure in SINQ Target-4.

For this purpose a SINQ Target Irradiation Program, STIP [12], has been started already with the first SINQ targets [6]; see Figure 16. Nearly ten thousand test specimens of various materials were irradiated in eight SINQ targets in the last two decades. Since 1998 STIP has been the unique international irradiation program oriented for spallation target applications.

NEURAP has been one of important facilities used for STIP. Essentially all the rods with test specimens were inspected before and after irradiation. Because of high activity and some other limitations, the post-irradiation examinations (PIE) are difficult or even impossible to be performed on some specimens in PSI’s hot laboratory. In such cases, NEURAP is the only tool available for PIE. Figure 14 presents an example of such a case showing neutron radiographies of a rod with several tens of specimens irradiated in three double-walled Hg capsules. The irradiation (STIP-II [8]) was conducted in years 2000 and 2001 for the purposes to understand materials behaviors in a Hg (the target material of SNS and JSNS) environment under irradiation. The upper radiograph depicts the poor wetting of Hg to the inner specimens (of ferritic and austenitic steels) and the wall of the capsule (of SS316L). After irradiation, the Hg spread rather homogeneously in the capsules, reflecting a good wetting. Furthermore, it can be observed that the Hg in the middle capsule leaked out and filled the gaps in the rod. In another rod (Figure 16) of the same irradiation experiment, many samples were irradiated in contact with Pb-Bi eutectic, the target material of MEGAPIE [13]. In this rod, a big capsule of the martensitic steel T91 contained many tensile specimens and TEM discs (used for transmission electron microscopy measurements). They are clearly seen the graph taken before irradiation. After irradiation, they are still well visible in the images. These results indicate a stronger corrosion effect of Hg on steels as compared with that of PbBi-eutectic in irradiation condition.

NEURAP is essential for this type of investigations; to the best of our knowledge, no similar possibilities exist elsewhere. 

## 4. Conclusions and Outlook

NEURAP, the dedicated installation at the SINQ spallation source of PSI operated as an add-on to the neutron-imaging instrument NEUTRA, offers unique possibilities for investigations of highly radioactive specimen. These capabilities proved to be very useful in the past for a wide range of demanding topics such as fuel pin enrichment, burn-up, and integrity on the level of single fuel pins and pellets. 

For SINQ itself, in particular, for the highly optimized SINQ target, NEURAP has demonstrated to be an indispensable tool to optimize performance while guaranteeing a safe and reliable operation. In cases where there were problems, NEURAP investigations gave most valuable results to avoid similar problems in later targets and continue optimization. 

The methodology using the activation of Dysprosium to separate neutron images from background generated from a sample itself, has continuously been improved over the years and still offers further possibilities for enhancements. 

We are aware of other “self-exposure techniques” using CsI samples [14] in combination with CCD chips. Unfortunately, the field of view (FOV) and the spatial resolution does not fit to our requirements. 

A current theme for further development of the method focuses on Dy-doped scintillators to be imaged by digital cameras. Devising special mechanics, which allow for fast movement of the scintillator out of the beam could allow for harnessing the fast decay channel Dy-165. At the same time, this would drastically reduce the time required per image, thus allowing for tomographic investigations of highly radioactive samples. Preliminary attempts for tomographic imaging [15] based on a very limited number of projections, could be enhanced to tomographic acquisitions of radioactive specimens with the number of projections close to meeting the requirements for the relevant sampling theorem could thus be become feasible [14].

Samples with limited dimensions and radioactivity such as defueled cladding sections can also be investigated with the neutron microscope detector [16] at the SINQ beamlines, using the recently developed Active Box sample container [17]. This box ensures the contamination free transport and installation at the beam line. However, samples with adherent fuel cannot yet be investigated using this tool. A step ahead will be an optimization of NEURAP so that fuel rod sections can be comparably well visualized with sufficiently high spatial resolution.

## Data Availability

There are no data published.
